# Nanomedicine-based combination therapies for overcoming temozolomide resistance in glioblastomas

**DOI:** 10.20892/j.issn.2095-3941.2022.0761

**Published:** 2023-05-05

**Authors:** Chun Wang, Qiushi Li, Jian Xiao, Yang Liu

**Affiliations:** 1 State Key Laboratory of Medicinal Chemical Biology, Nankai University, Tianjin 300071, China; 2 Key Laboratory of Functional Polymer Materials of Ministry of Education, College of Chemistry, Nankai University, Tianjin 300071, China; 3 Frontiers Science Center for New Organic Matter, Nankai University, Tianjin 300071, China

**Keywords:** Combination therapy, drug resistance, glioblastoma, nanotechnology, temozolomide

## Abstract

Glioblastoma (GBM) is the most common malignant brain tumor. Although current treatment strategies, including surgery, chemotherapy, and radiotherapy, have achieved clinical effects and prolonged the survival of patients, the gradual development of resistance against current therapies has led to a high recurrence rate and treatment failure. Mechanisms underlying the development of resistance involve multiple factors, including drug efflux, DNA damage repair, glioma stem cells, and a hypoxic tumor environment, which are usually correlative and promote each other. As many potential therapeutic targets have been discovered, combination therapy that regulates multiple resistance-related molecule pathways is considered an attractive strategy. In recent years, nanomedicine has revolutionized cancer therapies with optimized accumulation, penetration, internalization, and controlled release. Blood-brain barrier (BBB) penetration efficiency is also significantly improved through modifying ligands on nanomedicine and interacting with the receptors or transporters on the BBB. Moreover, different drugs for combination therapy usually process different pharmacokinetics and biodistribution, which can be further optimized with drug delivery systems to maximize the therapeutic efficiency of combination therapies. Herein the current achievements in nanomedicine-based combination therapy for GBM are discussed. This review aimed to provide a broader understanding of resistance mechanisms and nanomedicine-based combination therapies for future research on GBM treatment.

## Introduction

Glioblastoma (GBM), classified as a grade IV glioma, is the most common and aggressive adult brain tumor, accounting for 50% of all gliomas^1^. The standard GBM treatment involves maximal surgical resection, followed by postoperative radiotherapy and adjuvant chemotherapy with temozolomide (TMZ)^2,3^, the first-line chemotherapeutic drug for GBM treatment approved by the Food and Drug Administration (FDA). The recurrence rate of GBM, however, is as high as 90% due to incomplete tumor removal and development of resistance^4^. Although numerous clinical trials have demonstrated that patient survival is significantly prolonged with standard treatment^5^, the median survival of patients with GBM is < 2 years, and only approximately 5% of patients survive > 5 years^6^.

**Figure 1:**
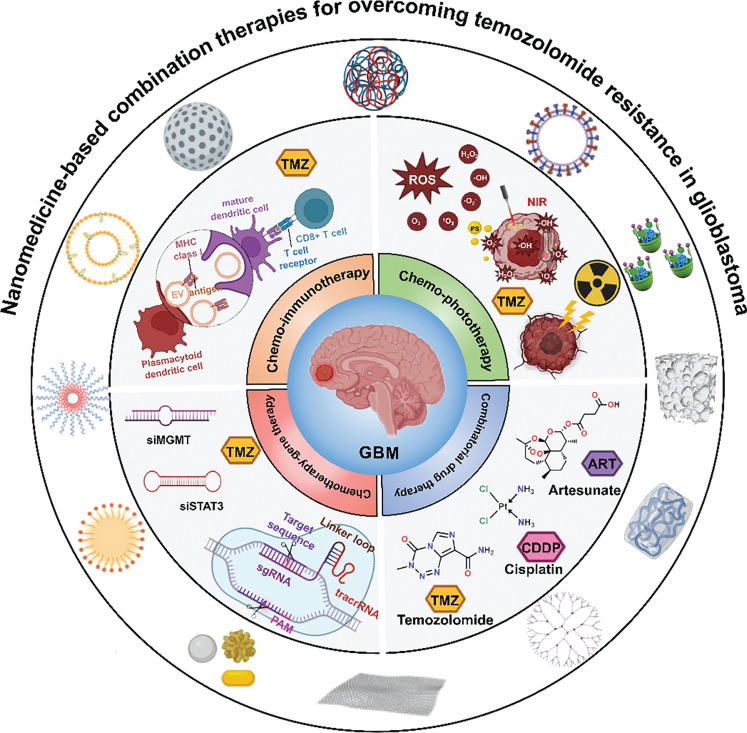
Schematic illustration of nanomedicine-based combination therapies for overcoming GBM drug resistance.

Compared to other malignant tumors, the blood-brain barrier (BBB) presents a unique and challenging biological barrier to effective drug delivery for GBM treatment^7,8^. Thus, commonly used chemotherapeutic drugs are not used in GBM treatment due to poor penetration across the BBB. Specifically, epirubicin, a structural analog of the anthracycline, doxorubicin, exhibits prominent cytotoxic against GBM tumor cells *in vitro*, but cannot serve as a clinical medication due to poor BBB permeability^9,10^. Although TMZ exhibits a distinct advantage in crossing the BBB, accumulation of TMZ in GBM is far from satisfactory. TMZ has been reported to be one of the substrates of P-glycoprotein (P-gp), which serves as an efflux pump on the apical membrane side of the endothelial cells forming the BBB^11,12^. Only 20% of the dose of TMZ enters the brain^13^. As a benefit of nanotechnology, chemotherapeutic drugs can be loaded into nanocarriers to improve BBB penetration efficiency through various transcytosis pathways^8^. GBM, however, is highly heterogeneous and prone to mutate^14^, thus providing a favorable environment to develop resistance to chemotherapeutic drugs. Considering the complicated signal networks and various compensatory mechanisms involved in the development of drug resistance, combination therapies that synergistically regulate multiple cancer-associated pathways using different treatments or drugs provide a promising strategy for overcoming drug resistance^15,16^. Further, nanotechnology optimizes the *in vivo* pharmacokinetics and biodistribution between multiple types of drugs, including small molecule drugs, therapeutic genes, and proteins, leading to improvement in the therapeutic efficiency and alleviation of side effects^17^. In this review we discuss mechanisms of drug resistance in patients with GMB and a possible strategy to overcome drug resistance. Then, we discuss the essential role of nanotechnology in combination therapies, outline current nanomedicine-based combination therapies for GBM treatment (**Figure 1**), and analyze the challenges to offer a reference for future research.

## GBM drug resistance mechanisms

There are two major approaches by which tumor cells develop drug resistance: 1) impeding the accumulation of chemotherapeutic drugs within tumor cells; and 2) developing DNA repair mechanisms to lower drug sensitivity^18^. In addition, the highly heterogeneous and hypoxic environment of GBM provides favorable conditions for developing drug resistance. This section focuses on common resistance mechanisms and potential strategies to improve chemosensitivity.

### Reduce drug uptake and increase drug efflux

Most chemotherapeutic drugs are only active inside tumor cells. Thus, to impair chemotherapeutic efficacy, tumor cells usually develop drug resistance by blocking or limiting access of the drug at the site of action^19^. For example, the expression of a series of solute carriers (SLCs) is downregulated. Thus, the uptake of drugs depending on SLCs to effect intracellular transportation is significantly decreased^18^. To overcome resistance caused by this mechanism, drugs can be loaded into delivery systems and enter tumor cells through alternative transport pathways.

Drug molecules must remain in the tumor cell at a sufficient concentration rather than pass through the cell to achieve effective treatment. ATP-binding cassette (ABC) transporter family proteins, including P-gp, multidrug resistance proteins (MRPs), and brain cancer resistance proteins (BCRPs), are usually overexpressed on tumor cell membranes and play an important role in drug resistance^20^. In a previous report, nearly 60% of chemotherapeutic drugs were recognized by P-gp, then transported outside the cell^21^, thus leading to decreased intracellular drug accumulation and diminished therapeutic activity. During GBM treatment, increased P-gp levels have been reported from tissue samples of clinical patients who exhibit TMZ resistance and tumor recurrence^22^. Knockdown of *MDR1*, which codes P-gp in GBM cells, results in enhanced TMZ-mediated cell death, thus suggesting that the *MDR1* gene has an important role in TMZ resistance^23^. Moreover, extracellular vesicles (EVs) usually contain mRNAs, miRNAs, and lncRNAs related to drug efflux, which are released from resistant cells and transferred to sensitive cells, further resulting in acquired resistance of surrounding cells within the tumor mass. To overcome resistance caused by this mechanism, small molecular inhibitors, such as P-gp inhibitors and small interfering RNA (siRNA) targeting ABC efflux gene families, have been designed to reduce drug efflux and alleviate TMZ resistance.

### DNA damage repair

The mechanism by which numerous chemotherapeutic drugs cause apoptosis of tumor cells is to induce DNA damage. TMZ, an oral alkylating agent, usually causes DNA damage by alkylating or methylating DNA at the O^6^ position of guanine residues^24^; however, under prolonged treatment, cancer cells gradually develop DNA repair mechanisms that reduce chemosensitivity. O^6^-methylguanine methyl transferase (MGMT), a DNA repair enzyme that eliminates the methyl group in O^6^-methylguanine and repairs drug-induced DNA damage, is considered to be a major factor in the development of TMZ resistance. Thus, TMZ usually fails to realize the expected therapeutic effect for GBM in patients with high MGMT expression. Moreover, MGMT expression increases with the development and recurrence of GBM^25^, thus leading to difficulties in the treatment of patients in the mid and late stages, as well as the relapse stage, which significantly affects the therapeutic effect. The use of a high dose of TMZ is the currently recommended strategy to counteract the attenuating effect of MGMT on DNA alkylation^26^; however, this maneuver does not overcome drug resistance and can even exacerbate drug resistance. To this end, DNA repair inhibitors, such as O^6^-benzylguanine (BG) and siRNA-targeted MGMT, have been developed to alleviate TMZ resistance based on the DNA damage repair mechanism.

### Heterogeneous tumor microenvironment

#### Glioma stem cells (GSCs)

Cancer stem cells (CSCs) are a population of undifferentiated and highly tumorigenic cancer cells that exhibit proliferative and self-renewal properties. While GSCs account for as few as 3%–5% of GBM tumors^27^, GSCs are a dominant factor in cancer progression, metastasis, and relapse, as well as the development of drug resistance^28^. Thus, although the initial treatment kills the bulk of tumor cells, a small number of residual GSCs survive and account for recurrence and treatment failure. Moreover, Liu et al.^29^ reported that the expression of ABC transporters is elevated in patients with recurrent GBM compared to patients with newly diagnosed GBM, which manifests as more severe drug resistance in recurrent GBM and treatment difficulty.

To develop promising therapies for elimination of GSCs, it is essential to understand the intrinsic signaling pathways responsible for the growth, renewal, and development of GBM. For example, Notch signaling is activated in GSCs, thus suppressing differentiation and maintaining stem-like properties, further contributing to tumorigenesis and therapeutic resistance^29^. Sonic hedgehog (SHH) signaling also has a vital role in regulating GSC self-renewal and tumorigenesis^30^. Moreover, it has been reported that inhibition of the SHH pathway sensitizes GSCs to TMZ treatment, providing a possible direction for overcoming TMZ resistance^31^. In addition, the Wnt/β-catenin pathway involves upregulation of MGMT expression, thus inducing the development of TMZ resistance in GBM^32^. To achieve effective treatment of GBM, novel therapeutic approaches are required to target the tumor bulk, and more importantly, to aim at GSCs to overcome resistance mechanisms.

#### Hypoxia and autophagy

Hypoxia is one of the characteristics of glioblastoma tissues. Hypoxia leads to an increase in genetic mutations, inflammation, the epithelial-mesenchymal transition (EMT), drug resistance, and autophagy. Musah-Eroje and Watson^33^ reported the interrelationship between hypoxia and TMZ resistance of GBM cells using a basement membrane extract (BME)-based 3D model. Hypoxia-inducible factor-1alpha (HIF-1α) is a crucial transcription factor. HIF-1α is significantly upregulated in the hypoxic microenvironment, which further leads to the activation of several genes associated with the hypoxia-responsive element (HRE), including the *MDR1* gene, one of the genes related to drug resistance^34^.

Autophagy, an important homeostatic cellular recycling mechanism, has a cytoprotective role by which excessive or unnecessary proteins and injured or aged organelles are degraded. Autophagy is usually activated after chemotherapy or radiotherapy to protect tumor cells from DNA damage, and thus is considered an important factor in the development of drug resistance^35^. For example, acute treatment with TMZ inhibits PI3K/Akt-mTOR and causes the transient induction of autophagy, further leading to TMZ resistance in GBM therapy. To solve this problem, chloroquine (CQ) and CQ analogs, which have been reported to inhibit autophagy, are used in clinical trials preceding treatment with TMZ.

The mechanisms underlying drug resistance in patients with GBM are complex and interconnected. For example, a hypoxic tumor environment with poor vasculature density is a hotbed for GSCs, which increase the levels of GSC marker expression and promote a cancer stem-like phenotype^36^. Persano et al.^37^ reported that silencing HIF-1α expression reduces MGMT-related drug resistance to TMZ, implying a positive relationship between HIF-1α and chemoresistance in GSCs. In addition, autophagy is usually upregulated in a hypoxic tumor environment with a lack of nutrition, further promoting GSC survival and migration, which are closely related to the development of chemoresistance^38^. Moreover, other potential pathways are still being discovered, which makes resistance mechanisms clear and increase the difficulties in overcoming resistance mechanisms.

## The role of nanotechnology in combination therapies

The rapid development in nanotechnology has provided new opportunities for cancer treatment. Multiple drug delivery systems (DDSs), including liposomes, polymers, and inorganic nanoparticles, have been designed to satisfy the diverse delivery requirements of different drugs. Current DDSs exhibit low immunogenicity, a high loading capacity, and preferentially accumulate in tumor tissues *via* an enhanced permeability and retention (EPR) effect^39^. Further, the accumulation, penetration, internalization, and controlled release of DDSs can be optimized through modulation of nanoparticle physicochemical and mechanical properties, including size^40^, shape^41^, surface chemistry^42^, and mechanical softness^43^. Moreover, BBB penetration efficiency has also been optimized with the development of nanotechnology. Through the receptors or transporters on the BBB, DDSs actively target brain tissues with high specificity, selectivity, and affinity. For example, glucose transporters (GLUTs) facilitate the transportation of glucose from the blood to the brain, which could be utilized to penetrate through BBB. Wu et al.^44^ used maltobionic acid, a glucose derivative, to modify a nanodelivery system, thus achieving enhanced BBB penetration efficiency. Angiopep-2 is a peptide targeting low-density lipoprotein receptor-related protein 1 (LRP1), which is often used to cross the BBB. Zheng et al.^45^ developed angiopep-2 ligand camouflaged polymeric siRNA nanomedicine, with which BBB penetration and GBM targeting was achieved. In addition, Fan et al.^46^ developed human H-ferritin (HFn) nanocarriers that successfully crossed the BBB through transferrin receptor 1 (TfR1)-mediated endocytosis, resulting in efficient tumor killing. Thus, introducing suitable ligands on DDS significantly improves BBB penetration efficiency.

For most combination chemotherapies, the ratio of the drugs or active ingredients in the combination is a crucial factor. Benefit from nanotechnology, pharmacokinetics, biodistribution, as well as the ratio of different drugs, are optimized to achieve better synergistic therapeutic efficacy. Xu et al.^47^ utilized mPEG-PLGA nanoparticles to co-deliver PTX and TMZ for GBM treatment. The best synergistic effect was achieved when the weight ratio of PTX-to-TMZ was 1:5 for U87 cells and 1:100 for C6 cells, demonstrating importance of the drugs ratio. The optimal drug ratio in cancer cells is usually different from the optimal drug ration *in vitro* due to the complicated tumor environment. To overcome this problem, Zhang et al.^48^ demonstrated a macrocyclic-amphiphile-based self-assembled nanoparticle (MASN) to precisely load multiple drugs for cancer treatment. Such macrocyclic host-based nanoparticles load drugs *via* host-guest interactions with a defined stoichiometry (most often 1:1), thus allowing multiple drugs to be precisely loaded with the optimal ratio. Moreover, owing to the sharp decline in the binding affinity as a response to the tumor environment, the MASN releases the loaded drugs simultaneously upon reaching tumors. Thus, with a ratiometric delivery ability of multiple drugs, a MASN has the potential to be an effective multi-drug delivery platform for combination chemotherapies of multiple cancers, including GBM.

Combinations of chemotherapy and gene therapy have also been successfully developed for cancer treatment. Small molecular drugs usually achieve anti-tumor effects by binding to cancer cell DNA and inducing DNA damage, which may interfere with the function of therapeutic genes, further leading to a compensation in the anti-tumor efficiency of both drugs and therapeutic genes. To solve this problem, Giese et al.^4^ first loaded small molecular drugs into carboxylated azocalix arene (CAC4A) *via* a host-guest interaction, which was further mixed with phenylboronic acid (PBA)-modified polyethyleneimine (PEI-PBA) and pDNA to form a calixarene-embedded polyplex core (denoted as CEPC)^49^. It is worth noting that CAC4A offers a steric barrier to avoid interference between the molecular drugs and the pDNA loaded in the CENP, thus enhancing the combined therapeutic efficacy and alleviating side effects. Considering the common feature of gene delivery for different cancers, this strategy is also expected to be used for GBM treatment. Therefore, through an elaborate design of DDS, it is possible to maximize the therapeutic efficiency of combination therapies.

## Nanomedicine-based combination therapies to overcome drug resistance

Because GBM resistance is complicated in multiple molecular pathways and the compensation mechanisms, inhibiting a single target is not sufficient to reverse resistance. Thus, combination therapy that targets multiple pathogenic pathways is an attractive strategy to address drug resistance and improve chemosensitivity of GBM. Recent evidence from clinical trials suggests that a combination of lomustine and TMZ improves overall survival when used as a first-line treatment for patients with MGMT methylation^50^. Also, the combined treatment of nimotuzumab, a monoclonal antibody against epidermal growth factor receptor (EGFR), and rapamycin, an mTOR inhibitor, achieved more efficient outcomes than TMZ treatment on patient-derived human glioma cells^51^. These results indicate the feasibility of combination treatment. In this section, we discuss multiple combination therapies, including combination chemotherapy, chemotherapy-gene therapy, chemo-phototherapy, and chemo-immunotherapy, to provide a reference for designing drug combinations and delivery systems for further research **(Table 1)**.

**Table 1:** Nanomedicine-based combination therapies for overcoming GBM drug resistance

Type	Formulations	Payloads	Mechanism	Cell lines	Refs
Combination drug therapy	Acetal-grated dextran; GBM cell membrane	TMZ; CDDP	Alleviating DNA damage repair	U87MG; U251-TR	^53^
Combination drug therapy	MPC nanocapsule	Inherbin3; cMBP	Alleviating DNA damage repair	U87MGR	^57^
Combination drug therapy	ApoE-functionalized liposomal nanoparticles	ARTPC; TMZ	Alleviating DNA damage repair	U251-TR	^59^
Combination drug therapy	Re-assembled exosomes	DHT; TMZ	Inhibiting drug efflux and DNA repair damage	GL261	^62^
Combination drug therapy	Transferrin-functionalized PEGylated nanoparticles	JQ1; TMZ	Regulating the DNA damage response	U87MG; GL261	^64^
Combination drug therapy	B19 aptamer-conjugated dendrimer nanoparticles	PTX; TMZ	Inducing GSCs apoptosis	U87 stem cells	^65^
Combination drug therapy	Gold nanoparticles	HCQ; DOX	Inhibiting cytoprotective autophagy	C6	^68^
Chemotherapy-gene therapy	Liposome-based nanoparticles	siMGMT; TMZ	Alleviating DNA damage repair	U87MG	^69^
Chemotherapy-gene therapy	Polymer-coated iron oxide nanoparticles	siMGMT; TMZ	Alleviating DNA damage repair	GBM6	^70^
Chemotherapy-gene therapy	BMSC exosomes	siSTAT3; TMZ	Alleviating DNA damage repair	U251-TR	^73^
Chemotherapy-gene therapy	Liposome and macrophage exosomes	si-c-Myc; si-mTOR; TMZ	Inhibiting GSC proliferation	U87; GC-4	^76^
Chemotherapy-gene therapy	PEI wrapped spherical nucleic acid nanoparticles	si Gli1; TMZ	Inhibiting self-renewal capacity of GSCs	U87MG	^78^
Chemotherapy-gene therapy	Mesoporous silica nanoparticles	R8-PNA; TMZ	miR221 downregulation	T98G	^79^
Chemotherapy-gene therapy	Polymer-based nanoparticles	DOX; miR-125b	Aggravating DNA damage	U251	^80^
Chemotherapy-gene therapy	Lipid-polymer hybrid nanoparticles	pCas9; MGMT	Alleviating DNA damage repair	T98G	^81^
Chemo-phototherapy	Pluronic P85/F127 nanoparticles	VP; TMZ	Circumventing drug resistance pathways	U87MG	^86^
Chemo-phototherapy	Polymer-based nanoparticles	VP; PTX	Circumventing drug resistance pathways	U87MG	^87^
Chemo-phototherapy	Gold nanoparticles	TMZ	Activating p53 and alleviating DNA damage repair	T98G	^88^
Chemo-phototherapy; HBO	Porous silicon nanoparticles	TMZ	Relieving the hypoxic microenvironment	C6	^89^
Chemo-phototherapy	Liposome-based nanoparticles	DCHB; TMZ-C18	Circumventing drug resistance pathways	U87MG	^90^
Chemo-immunotherapy	Lipid polymer nanoparticle	siPD-L1; TMZ	Reversing the tumor suppressive microenvironment; alleviating DNA damage repair	C6/TR	^91^
Chemo-immunotherapy	MnO_2_ nanoparticles; PEG-PAE	TMZ	Reversing the tumor suppressive microenvironment; relieving the hypoxic microenvironment	G422	^92^
Chemo-chemodynamic therapy	iRGD-modified polymeric micelles	TMZ; MnO	Relieving the hypoxic microenvironment	C6	^93^
Chemo-sonodynamic therapy	Gold nanoparticles	RA; TMZ	Inducing CSCs differentiation	U87MG	^95^

### Combination drug therapy

**Figure 2:**
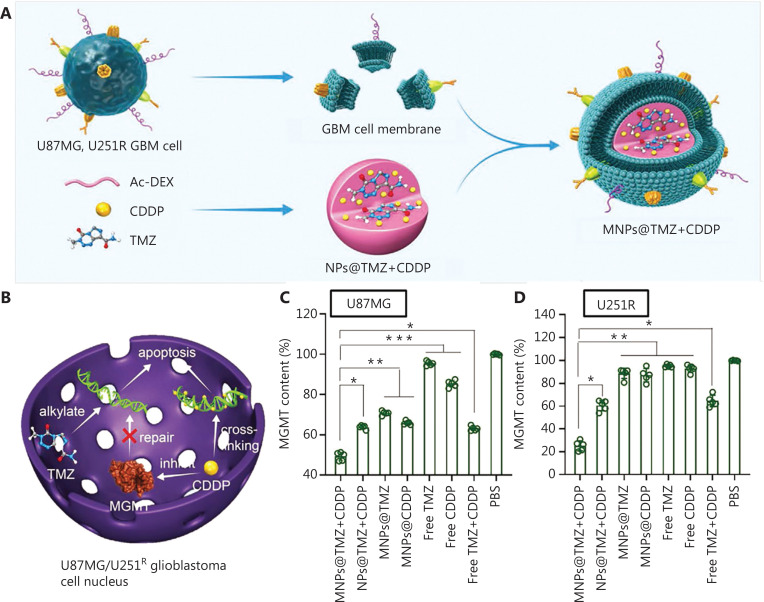
MNPs@TMZ+CDDP co-delivery of TMZ and CDDP for combination GBM chemotherapy. (A) Schematic for the preparation of MNPs@TMZ+CDDP. (B) Illustration of the mechanism underlying synergistic action of TMZ and CDDP in the cell nucleus. MGMT concentration (% PBS control) in U87MG (C) and U251R (D) cells after a 48-h incubation with MNPs@TMZ+CDDP, as assessed by ELISA. Data are presented as mean ± SD [*n* = 5, one-way analysis of variance (ANOVA) and Tukey multiple comparisons tests, *P < 0.05, **P < 0.01, ***P < 0.001]. Reprinted with permission from reference^53^. Copyright 2022, Wiley.

Combination drug therapy with two or more drugs to disturb multiple pathogenic pathways is the most direct strategy to address the challenge of drug resistance. Co-delivering drugs for reducing MGMT activity is the most common strategy. For example, cisplatin (CDDP), a common clinical anticancer drug for multiple cancers, binds DNA and causes crosslinking of purine bases, thereby destroying the function of DNA and inhibiting cell mitosis. Moreover, CDDP has also exhibited the ability to decrease MGMT activity, suggesting its potential in combination therapies with a DNA-damaging agent. In a phase II trial, the combination of CDDP and TMZ increased patient survival compared with TMZ alone^52^; however, the therapeutic effect of combination multiple chemotherapy drugs is limited by poor BBB penetration, limited tumor-targeting ability, and high systemic toxicity. To solve these issues with the assistance of nanomaterials, Zou et al.^53^ successfully developed a novel strategy for the co-delivery of TMZ and CDDP with a pH-responsive acetal grated dextran (AcDEX) core and GBM cell membrane (CCM, denoted MNPs@TMZ+CDDP; **Figure 2**). It has been reported that engineered nanoparticles with GBM CCM target homologous cancer cells due to homotypic recognition by the abundant proteins on the surface of CCM^54^. Moreover, because the GBM cell membrane readily traverses the BBB due to the downregulation of the specific proteins (ZO-1 and claudin-5) in tight junctions^55^, such biomimetic nanoparticles achieve efficient BBB transportation and GBM targeting. As a result, MNPs@TMZ+CDDP crosses the BBB and delivers both TMZ and CDDP into the tumor. Compared with single drug-loaded nanoparticles, MNPs@TMZ+CDDP extends survival up to 3-fold without noticeable side effects. The EGFR and mesenchymal-epithelial transition factor (MET) have been shown to be involved in TMZ resistance and GBM cell growth^56^. As two inhibitors, Inherbin3 and cMBP block their corresponding pathways, thus impairing DNA damage repair to inhibit drug resistance. To co-deliver Inherbin3 and cMBP, and regulate the activity of EGFR and MET, Meng et al.^57^ developed a dual functionalized brain-targeting nanoinhibitor (denoted BIP-MPC-NP) through *in situ* polymerization based on 2-methacryloyloxy ethyl phosphorylcholine (MPC), followed by the surface conjugation with Inherbin3 and cMBP. As a result, BIP-MPC-NP effectively decreased the activation of EGFR and MET, thereby attenuating DNA damage repair in drug-resistant GBM and enhancing the sensitivity of GBM to TMZ.

As a commonly used clinical Chinese medicine, artesunate (ART) involves multiple pathways within tumor cells. Recent research reported that ART interferes with the expression of MGMT and alleviates GBM drug resistance by inhibiting the Wnt/β-catenin pathway^58^. Based on ART, Ismail et al.^59^ employed artesunate-phosphatidylcholine (ARTPC) to encapsulate TMZ and constructed a special ApoE-functionalized liposomal nanoplatform (denoted ApoE-ARTPC@TMZ). Such a nanoplatform enhanced GBM sensitivity to TMZ and achieved great combination chemotherapy for resistant tumors. Similarly, dihydrotanshinone (DHT), another traditional Chinese medicine, induces glioma cell apoptosis by upregulating caspase-3 expression and reduces drug resistance by downregulating MGMT and P-gp expression^60,61^. Wang et al.^62^ utilized glioma cell-derived reassembled exosomes to co-deliver TMZ and DHT (denoted R-EXO-T/D). The results showed that the combination of DHT and TMZ significantly enhanced the anti-glioma effect of TMZ in an orthotopic GL261-bearing mice model by inhibiting drug efflux and DNA repair damage.

Floyd et al.^63^ reported that the potent small molecule inhibitor, JQ1, inhibited the expression of BET bromodomain proteins, further regulating the DNA damage response in multiple cancer cell lines, including U87MG cells. In IC50 analyses of U87MG and GL261 cells, the combinatorial index (C.I.) values of TMZ and JQ1 were 0.95 and 0.94 for U87MG and GL261 cells, respectively, indicating an additive cytotoxic effect. Moreover, increased DNA damage was observed from the groups treated with the combination of JQ1 and TMZ. Further, Lam et al.^64^ utilized transferrin-functionalized PEGylated NPs (Tf-NPs) to co-deliver JQ1 and TMZ, penetrate the BBB, and target GBM. *In vivo* experiments demonstrated that JQ1 and TMZ co-loaded Tf-NPs significantly sensitize gliomas to TMZ therapy, resulting in effective tumor growth inhibition in both U87MG- and GL261-bearing mice.

In addition, co-delivering the drugs for regulating DNA damage or inhibiting drug efflux, specifically inducing GSCs apoptosis, is another practical approach. Behrooz et al.^65^ designed B19 aptamer-conjugated dendrimer nanoparticles to co-deliver paclitaxel (PTX) and TMZ (denoted Apt-NPs). Owing to the high affinity between B19 aptamer and CD133, a most recognizable biomarker on GSCs, Apt-NPs target U-87 stem cells and induce apoptosis, further reducing the resistance in GBM.

It has also been reported that inhibiting cytoprotective autophagy improves chemosensitivity in GBM^66^. CQ and its derivative [hydroxychloroquine (HCQ)] are lysosomotropic agents that increase lysosomal pH, thereby blocking autophagosome fusion with the lysosome^67^. Ruan et al.^68^ designed functional GNPs to co-deliver DOX and HCQ for chemotherapy and autophagic interference. As expected, the released HCQ inhibited the DOX-induced cytoprotective autophagy and re-sensitized glioma cells to DOX, thus providing an effective strategy for overcoming drug resistance.

### Chemotherapy-gene therapy

In addition to combination chemotherapy, gene therapy, which directly disturbs disease-causing genes or drug-resistant genes, is another method to overcome tumor drug resistance. Gene therapy refers to a treatment that utilizes gene editing tools to down-regulate or up-regulate, replace, or insert specific genes to alleviate the development of diseases. For example, using siRNA to downregulate the overexpression of MGMT in GBM cells effectively enhances the sensitivity of GBM to TMZ and alleviates the drug resistance of GBM.

**Figure 3:**
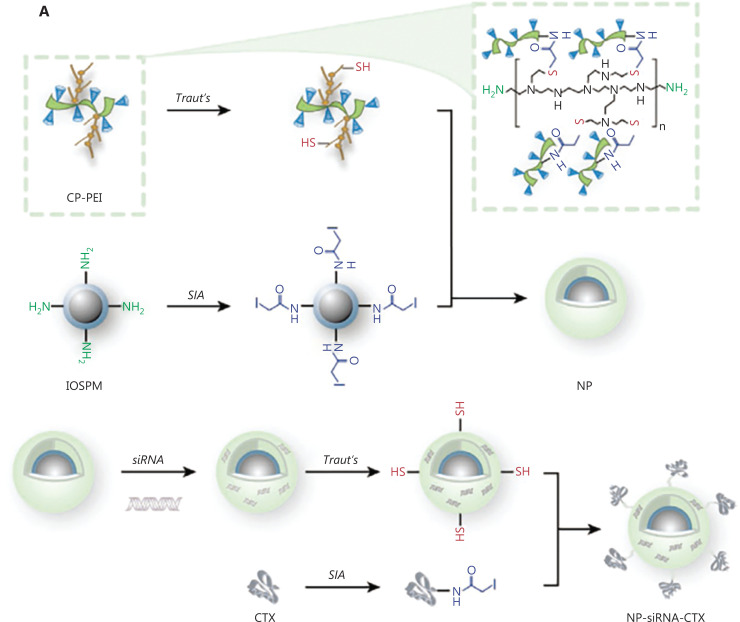

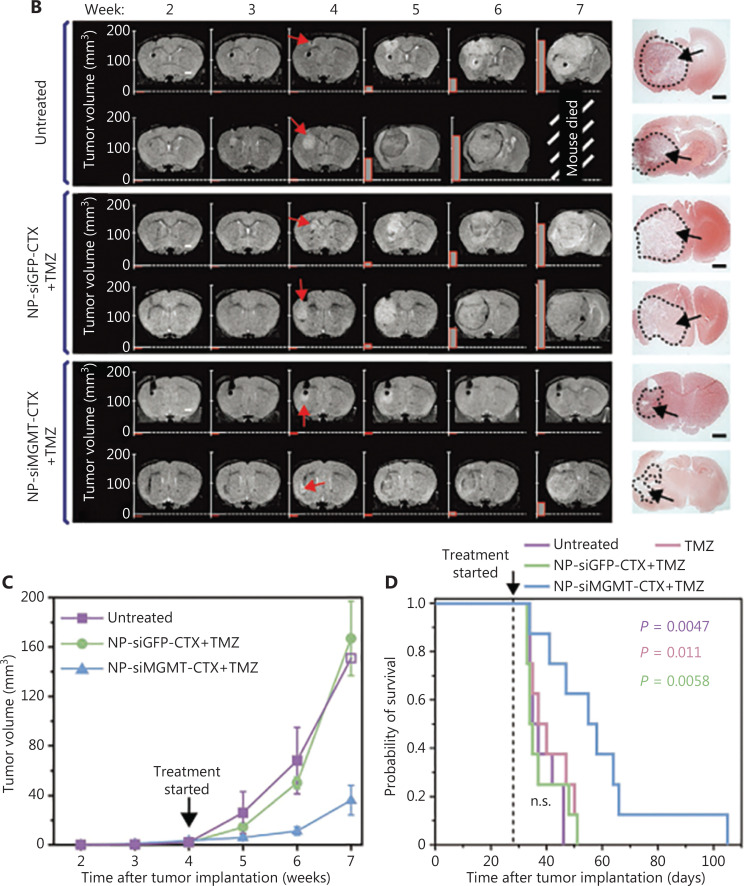
Delivery of siMGMT for TMZ-resistant glioblastoma therapy. (A) Schematic illustration for the synthesis of NP-siRNA-CTX. T2-weighted MRI images (B), tumor volumes (mean ± SD) in mice (C) and Kaplan−Meier survival curves (D) of different treated groups^70^. Copyright 2021, Wiley.

The instability of gene editing tools (nucleic acid or protein) in blood circulation and the difficulty of penetrating the BBB and entering GBM cells across amphiphilic cell membranes limit their application in regulating drug resistance of GBM. With the development and advances in nanotechnology, nanomaterials are emerging as ideal non-viral vectors capable of overcoming multiple biological barriers of nucleic acid delivery to GBM. For example, Xie et al.^69^ designed an attractive liposome-based hypoxia-radiosensitive nanoparticle nanoplatform denoted RDPP(Met)/TMZ/siMGMT for co-delivery of TMZ and siMGMT. Xie et al.^69^ encapsulated TMZ into RDPP by hydrophobic interaction and attached siMGMT to the surface of RDPP *via* an electrostatic interaction. Furthermore, surface modification of RGD facilitated RDPP to transport TMZ and siMGMT across the BBB and targeted GBM. Owing to the hypoxia-triggered degradation of RDPP, TMZ and siMGMT were released in the tumor microenvironment. Because siMGMT significantly decreased the expression of MGMT and restored the sensitivity of GBM to TMZ, the results showed that RDPP(Met)/TMZ/siMGMT effectively inhibited GBM proliferation and prolonged the survival of mice in an U87MG brain tumor mouse model. Similarly, Wang et al.^70^ developed chitosan-PEG-polyethylenimine (PEI)-coated iron oxide nanoparticles for siMGMT loading, followed by targeting ligand (CTX) conjugation (denoted NP-siRNA-CTX; **Figure 3**). Such nanoparticles reduced MGMT expression in both GBM cells and GSCs, thus improving TMZ chemosensitivity and prolonging the survival of mice bearing orthotopic GBM6 tumors. In addition, recent research has shown that the signal transmitter and activator of transcription 3 (STAT3) is one of the genes that regulates cell proliferation and anti-apoptosis in various tumor cells. STAT3 induces the expression of MGMT in GBM cells, leading to the development of drug resistance^71,72^. Rehman et al.^73^ employed siRNA to silence the expression of STAT3 in GBM to relieve the resistance of GBM to TMZ. Based on exosomes isolated from bone marrow mesenchymal stem cells (BMSCs), Rehman et al.^73^ constructed a multifunctional nanocarrier to deliver TMZ or siSTAT3, followed by modification with HMOX1-specific peptide (HSSP), which targets GBM transport (denoted HSSP-BMSC_Exo_). As a result, HSSP-BMSC_Exo_ effectively crossed the BBB, silenced STAT3, restored GBM sensitivity to TMZ, and eventually inhibited tumor proliferation in a TMZ-resistant U251 (U251-TR) brain tumor mouse model.

c-Myc is also commonly activated in various CSCs, and the upregulation of mTOR contributes to c-Myc expression^74,75^. Ma et al.^76^ constructed a biological camouflaged nanosystem based on liposome and macrophage exosomes to co-deliver si-c-Myc, si-mTOR, and TMZ. Through effective downregulation of c-Myc and mTOR expression, the proliferation of GSCs was significantly suppressed, thus attenuating TMZ resistance. Such a dual-targeting inhibition strategy efficiently inhibited tumor growth and prolonged survival in TMZ-resistant GBM-bearing mice. In addition, the Hedgehog (Hh)/Gli1 signaling pathway maintains the proliferation and survival of GBM cells and GSCs^77^. Downregulation of Gli1 expression with RNAi technology suppresses GSC proliferation and reduces chemoresistance. Melamed et al.^78^ developed polyethylenimine-wrapped spherical nucleic acid nanoparticles (PEI-SNAs) as a siGli1 delivery carrier. With enhanced cellular uptake and endosomal escape, Gli1 PEI-SNAs effectively silenced Gli1 expression and inhibited the self-renewal capacity of GSCs. Thus, Gli1 PEI-SNAs significantly relieved TMZ resistance and induced effective cell apoptosis under low doses of TMZ.

Recently, more and more reports have suggested that the abnormal expression profile of miRNAs is closely related to cancer cell drug resistance. Downregulation of miR221 expression is beneficial in restoring the sensitivity of GBM cells to TMZ. As a commonly used anti-miR targeting tool, peptide nucleic acids (PNAs) have been shown to effectively target miRNAs. Bertucci et al.^79^ developed mesoporous silica nanoparticles (MSNPs) to co-deliver miR221-targeting polyarginine-peptide nucleic acid (R8-PNA) and TMZ (denoted PNA-TMZ-MSNPs). The *in vitro* results showed that PNA-TMZ-MSNPs effectively induced an apoptosis rate up to 70.9% in a TMZ-resistant T98G cell line. It has been reported that overexpression of miR-125b enhances the sensitivity of the DOX-resistant breast cancer cell line, MCF-7/R, to DOX and exhibits a similar potential in other tumors, including GBM. Wang et al.^80^ encapsulated DOX and miR-125b into an amphiphilic co-polymer (γ-PGA-co-PLA-DPPE) containing poly [gamma-glutamic acid (γ-PGA)], polylactide (PLA), and 1,2-dipalmitoyl-sn-glycero-3-phosphoethanolamine (DPPE), and formed co-polymer NPs using a nano-precipitation method. The results showed that miR-125b aggravates cell cycle arrest caused by DNA damage by DOX and activates the AMPK/p53 pathway in U251 cells, thus improving chemosensitivity and promoting tumor cell apoptosis.

As a revolutionary gene editing tool, the CRISPR/Cas9 system has also been used to overcome challenges in GBM drug resistance. For example, Yang et al.^81^ developed microbubble-modified lipid-polymer hybrid nanoparticles (denoted MB-LPHNs-cRGD) for efficient delivery of dual-gRNA/Cas9 plasmids targeting the MGMT gene (pCas9/MGMT). With focused ultrasound (FUS)-assisted BBB opening, MB-LPHN_spCas9_/MGMT-cRGD was effectively transported to GBM cells, thus relieving the drug resistance of GBM through *MGMT* downregulation. Finally, MB-LPHNs-cRGD significantly inhibited tumor growth and prolonged survival of T98G tumor-bearing mice.

### Chemo-phototherapy

Phototherapy, including photodynamic therapy (PDT) and photothermal therapy (PTT), as a non-invasive, highly selective, and controllable strategy, has attracted more and more attention in cancer treatment^82^. PDT employs photosensitizers (PSs) to absorb light and convert energy to cytotoxic ROS or heat to induce tumor cell apoptosis. In addition to inducing apoptosis, PDT also exhibits the ability to circumvent drug resistance, providing an important strategy to overcome GBM resistance^83,84^. Similarly, PTT induces tumor cell apoptosis directly *via* heat generation^85^. Thus, combining phototherapy with TMZ is expected to significantly enhance the tumor suppressive effect.

As an important element in phototherapy, PS converts light into ROS or heat; however, the limited BBB crossing and tumor penetration efficiency in GBM severely restrict the therapeutic effect of phototherapy combined with chemotherapy in overcoming tumor drug resistance. Recently, with the rapid development of nanotechnology, nanomaterials with the characteristics of small particle size, easy surface modification, and strong permeability provide solutions to the problems. For example, Pellosi et al.^86^ constructed multifunctional pluronic P85/F127 nanoparticles with biotin modification (m-NPs) to co-load TMZ and PS verteporfin (VP) for chemotherapy combined with PDT for GBM treatment. In addition to the excellent drug-loading ability, pluronics reduce drug efflux by inhibiting P-glycoprotein expression. As a result, *in vitro* experiments showed that VP/TMZ-coloaded mNP is more effective than VP-loaded m-NP or TMZ-loaded m-NP treatment alone, thereby proving that PDT combined with low-dose TMZ effectively suppresses tumor growth and overcomes the resistance by circumventing the drug resistance pathway. Similarly, Zhang et al.^87^ also constructed an angiopep-2 (Ang) peptide-modified multifunctional nanocarrier (denoted T-^TK^NP_VP_) to co-load VP and PTX. Under X-ray irradiation, T-^TK^NP_VP_ produces cytotoxic ROS and releases PTX. As a result, T-^TK^NP_VP_ significantly inhibits tumor proliferation and prolongs mouse survival in a U87-MG brain tumor mouse model through combination X-ray-induced PDT and chemotherapy.

**Figure 4:**
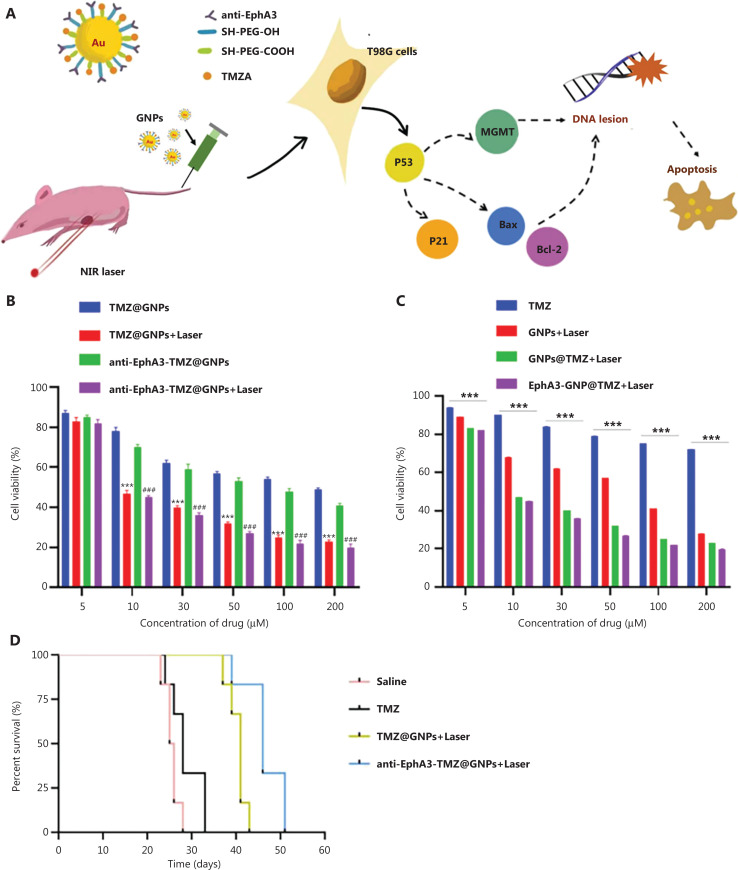
Delivery of TMZ-conjugated gold nanoparticles for photothermal therapy of drug-resistant glioblastoma. (A) Schematic illustration for the preparation of TMZ-conjugated gold nanoparticles and the associated mechanism. (B) Cytotoxicity of different treated groups for 48 h in T98G cells (*n* = 6), ***P < 0.001 versus TMZ@GNPs, ###*P* < 0.001 versus anti-EphA3-TMZ@GNPs. (C) Cytotoxicity of different treated groups for 48 h in T98G cells (*n* = 6), ***P < 0.001 versus TMZ. (D) Kaplan−Meier survival curves of different treated groups (*n* = 10)^88^. Copyright 2022, American Chemical Society.

In addition to PDT combined with chemotherapy, PTT has also been combined with chemotherapy to overcome drug resistance in GBM. For example, Yu et al.^88^ designed TMZ-loaded GNPs with anti-EphA3 modification on the surface (denoted anti-EphA3-TMZ@GNPs) to target GBM cells (**Figure 4**). Under laser irradiation, anti-EphA3-TMZ@GNPs enhance tumor cell apoptosis through a chemophotothermal synergistic effect. Moreover, such chemophotothermal treatment activates p53 and reduces *MGMT* expression. As a result, anti-EphA3-TMZ@GNPs effectively inhibit T98G glioma tumor proliferation and reverse GBM drug resistance. Zeng et al.^89^ designed porous silicon nanoparticles to load TMZ (denoted TMZ/Psi NPs) and achieved chemo-PTT and hyperbaric oxygen (HBO) therapy. Upon exposure to NIR light, Psi generates mild heat for thermotherapy (40-44°C), thus enhancing the drug effects. Moreover, HBO relieves the hypoxic microenvironment in GBM and increases TMZ sensitivity. As a result, TMZ/Psi NPs presents significant antitumor effects in C6 tumor-bearing mice with the combination of chemotherapy, PTT, and HBO.

Moreover, Zhang et al.^90^ fabricated a multifunctional phototheranostic agent (denoted DTRGD NPs) for chemo/photodynamic/photothermal synergistic therapy. DTRGD NPs were constructed by co-encapsulating PS [dicysteamine-modified hypocrellin derivative (DCHB)] and octadecane-modified TMZ derivative (TMZ-C18) with DSPE-mPEG2000 and DSPE-PEG2000-cRGD. With the BBB crossing ability, DTRGD NPs significantly inhibit GBM tumor growth and alleviate GBM drug resistance by chemo/photodynamic/photothermal synergistic therapy.

### Chemo-immunotherapy

Cancer immunotherapy, which aims to activate the host immune system to recognize and destroy tumors, has emerged as a potential strategy for cancer treatment after surgery, chemotherapy, and radiotherapy. By targeting the immune system rather than the tumor itself, immunotherapy achieves precise recognition and killing of tumor cells in an antigen-specific manner. In the past few decades, cancer immunotherapies, including immune checkpoint inhibitors (ICIs), adoptive T cell transfer (ATC), and chimeric antigen receptor T-cell (CAR-T), have achieved great success and revolutionized cancer treatment.

**Figure 5:**
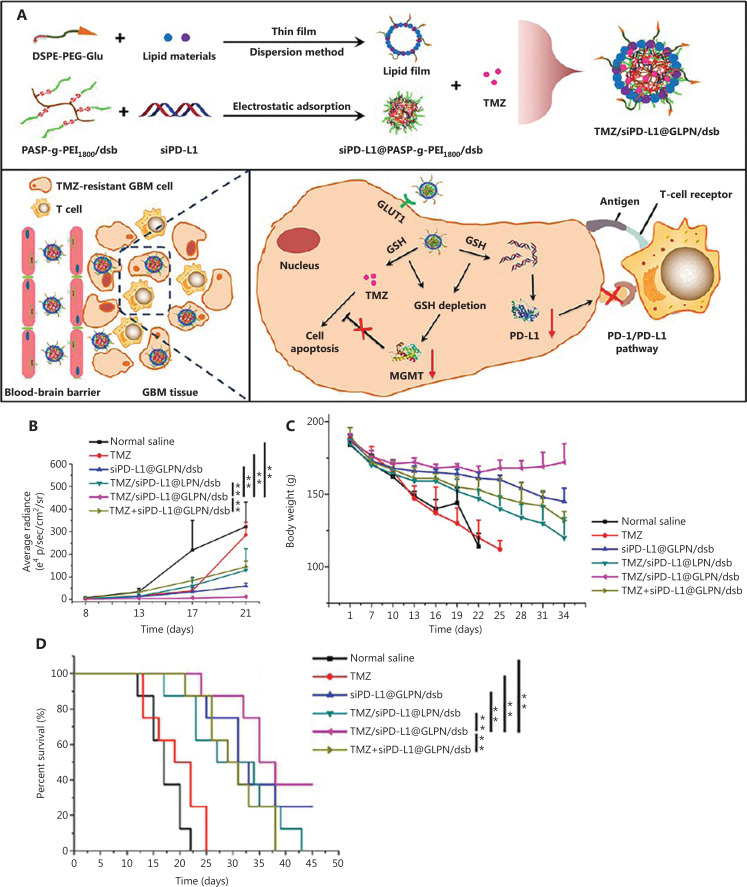
Co-delivery of TMZ and siPD-L1 to re-program the drug-resistant and immunosuppressive microenvironment in GBM. (A) Schematic for the preparation of TMZ/siPD-L1@GLPN/dsb and the mechanism underlying TMZ/siPD-L1@GLPN/dsb-mediated GBM therapy. (B) Bioluminescence intensity in the brain of different treated groups (*n* = 6, x̅ ± SD; **P < 0.01). (C) Body weight of different treated groups (*n* = 8, x̅ ± SD). (D) Survival curves of different treated groups (*n* = 8, x̅ ± SD; **P < 0.01). Reprinted with permission from reference^91^. Copyright 2022, American Chemical Society.

Therapeutic antibodies have been used to restore the activity of immune cells in GBM and reshape the tumor immunosuppressive microenvironment, which is closely related to GBM drug resistance. Recently, many clinical trials have utilized immune checkpoint blockade therapy combined with chemotherapy against GBM. Unfortunately, the results of these clinical trials are not satisfactory. Several studies have shown that the inability of therapeutic antibodies to cross the BBB limit their therapeutic efficacy against GBM. Over recent decades, with the rapid development of nanomaterials, various nanocarriers have been developed to overcome this issue. For example, Liu et al.^91^ designed a 2-deoxy-D-glucose modified lipid polymer nanoparticle to load TMZ and siPD-L1 (denoted TMZ/siPD-L1@GLPN/dsb) as combination chemotherapy and immunotherapy (**Figure 5**). Such nanoparticles downregulate the expression of PD-L1 in tumor cells, thus reversing the tumor suppressive microenvironment and reducing the activity of MGMT. With effective BBB penetration, enhanced GBM accumulation, and TMZ sensitivity, TMZ/siPD-L1@GLPN/dsb effectively inhibit GBM tumor proliferation and prolong the rat survival time in an orthotopic C6/TR brain tumor rat model. In another report, MnO_2_ nanoparticles were utilized to load TMZ, followed by surface modification of poly (ethylene glycol)-poly (β-amino ester) to improve stability (denoted MT@PAE)^92^. By increasing BBB permeability using ultrasound, MT@PAE and PD-L1 antibodies crossed the BBB, accumulated in GBM, and relieved the tumor immunosuppressive microenvironment. Moreover, MnO_2_ nanoparticles also relieved the hypoxic microenvironment by consuming excessive H_2_O_2_. As a result, the combination of MT@PAE and PD-L1 antibodies overcame GBM drug resistance and inhibited GBM tumor growth in a G422 brain tumor mouse model.

### Other combination therapies

Manganese dioxide (MnO_2_) and manganese oxide (MnO) nanoparticles have recently been used to modulate tumor hypoxia, which is considered an important factor to induce drug resistance. Tan et al.^93^ synthesized iRGD-modified polymeric micelles {polyethylene glycol-poly(2-(diisopropyl-amino) ethyl methacrylate, [PEG-PDPA]} to encapsulate TMZ and MnO (denoted iRPPA@TMZ/MnO). The released Mn^2+^ induced intracellular oxidative stress to cause tumor cell death *via* Fenton-like activity and the production of O_2_ further alleviated tumor hypoxia. Thus, such nanoparticles enhanced GBM tumor inhibition efficiency and alleviated GBM drug resistance *via* synergistic chemodynamic therapy and chemotherapy.

Sonodynamic therapy has been commonly used in cancer therapy, which not only enhances cancer cells apoptosis, but also regulates the tumor microenvironment. Moreover, low-intensity ultrasound (LIUS) has exhibited the ability to induce CSC attachment and differentiation^94^, thus reducing CSC stemness and drug resistance. Fadera et al.^95^ utilized GNPs as nano-sonosensitizers to enhance the ultrasound stimulation and load TMZ. In addition, retinoic acid (RA) has been widely reported to induce the differentiation of CSCs by blocking off signaling pathways. In this study the combination of RA and TMZ-loaded GNP-associated LIUS stimulation exhibited a significant and synergistic effect on promoting CSCs differentiation and further enhancing TMZ sensitivity.

## Discussion

Treatment of GBM remains a challenge, and TMZ resistance is one of the major factors in treatment failure. Although this review summarized resistance mechanisms simply as reduced drug uptake, increased drug efflux, DNA damage repair, and heterogeneous tumor microenvironment, more-and-more studies have shown that TMZ resistance is mediated by multiple molecular pathways. For example, the overexpression of EGFR, galectin-1, and Mdm2, as well as the mutation of p53 and phosphatase and tensin homolog (PTEN), have essential roles in drug resistance, providing a number of potential targets for relieving drug resistance and increasing difficulties in overcoming drug resistance. Thus, therapeutic regimens aimed at a single target usually fail to achieve effective GBM treatment. Although combination treatments targeting multiple pathways exhibit significant advantages over single target treatment, GBM is likely to develop compensation mechanisms through unknown pathways owing to intrinsic heterogeneity. As advances in molecular biology research, more innovative therapeutic targets and inherent interrelationships and compensation mechanisms are discovered and identified, laying a foundation for drug discovery and the combination of various drugs or therapies for effective long-term GBM treatment.

Nanomedicine has revolutionized GBM therapies. Current delivery methods based on nanotechnology have significantly improved BBB permeability and GBM accumulation of therapeutic agents and reduced systemic toxicity. Moreover, nanoparticles designed to load multiple therapeutics exhibit great potential in achieving effective combination therapy. With increasing knowledge of GBM molecular pathways related to drug resistance and the further development of delivery strategies, nanomedicine can be further optimized to achieve more effective GBM treatment. In addition, most combination treatments in the current studies co-deliver multiple therapeutics without carefully designing and optimizing the synergistic effects between the drugs. Thus, future research should focus on the factors (e.g., drug ratio, and spatial and tempo control of the drug release) that affect the efficiency of drug combinations in the design of new treatments for overcoming drug resistance.
